# Predictive value of pretreatment peripheral blood N/CD4 and N/CD8 ratios for the efficacy of radiotherapy for esophageal cancer

**DOI:** 10.2478/raon-2025-0015

**Published:** 2025-03-19

**Authors:** Yu-Rong Jiang, Yu-Ting Su, Jing Hu, Yan Ding, Lu Wang, Zi-Yu Wang, Wan-Ying Sheng, Yi-Xu Fan, Liang-Mei Chu, Yu-Fei Yang, Yi Wen, Miao Han, Si-Yuan Zhou, Chun-Hua Dai, Xu Wang

**Affiliations:** 1 Department of Thoracic Oncology, Cancer Institute of Jiangsu University, Affiliated Hospital of Jiangsu University, Zhenjiang, P. R. China; 2 Department of Abdominal Oncology, Cancer Institute of Jiangsu University, Affiliated Hospital of Jiangsu University, Zhenjiang, P. R. China; 3 Department of Head and Neck and Comprehensive Oncology, Cancer Institute of Jiangsu University, Affiliated Hospital of Jiangsu University, Zhenjiang, P. R. China

**Keywords:** peripheral blood N/CD4 and N/CD8, esophageal cancer, radiotherapy, efficacy prediction

## Abstract

**Background:**

This study aimed to explore the predictive value of pretreatment peripheral blood immune cell subsets in analyzing the outcomes of patients who underwent radiation therapy for esophageal cancer at their first visit.

**Patients and methods:**

This study included 72 patients with esophageal cancer (EC) treated at Jiangsu University Hospital from December 2021 to December 2023. Among them, 48 were males and 24 were females, with a median age of 64 years (range: 52–98 years). Comprehensive clinical data, laboratory results, and imaging findings were collected to analyze survival differences. The log-rank test was used for univariate analysis to assess the sensitivity of these patients to radiotherapy. The statistically significant and clinically relevant factors identified from the univariate analysis were subsequently incorporated into a Cox proportional hazards regression model for multivariate analysis to investigate the associations between pretreatment peripheral blood immune cell subsets and patient survival.

**Results:**

Univariate Cox regression analysis revealed that the Eastern Cooperative Oncology Group (ECOG) score, CD4^+^ T-cell ratio, neutrophil-to-CD4^+^ T-cell ratio (N/CD4), neutrophil-to-CD8^+^ T-cell ratio (N/CD8), and neutrophil-to-B-cell ratio (N/B) were significantly correlated with survival outcomes in patients receiving radiotherapy for tumors. Furthermore, multivariate Cox regression analysis identified N/CD4^+^ T cells and N/CD8^+^ T cells as critical prognostic indicators for these patients. Receiver operating characteristic curve analysis was employed to evaluate the work characteristics of the subjects, resulting in area under the curve values of 0.763 for both N/CD4 and N/CD8. The analysis also revealed that the optimal cutoff values for N/CD4^+^ T cells and N/CD8^+^ T cells were 0.01053329 and 0.01184294, respectively.

**Conclusions:**

N/CD4 and N/CD8 have emerged as viable prognostic predictors for patients undergoing radiotherapy for EC, offering valuable insights for clinicians to strategize further treatment options. However, the retrospective nature of this study introduces potential bias in assessment, underscoring the necessity for large-scale, prospective, randomized controlled trials to substantiate and validate these findings.

## Introduction

Esophageal cancer (EC) is a prevalent gastrointestinal tumor. Global cancer statistics from 2020 revealed that there were 604,000 new cases of EC and 544,000 associated deaths, with incidence and mortality rates varying significantly across different regions.^1^ China has been identified as a high-incidence area, with approximately 150000 deaths annually. EC typically affects more males than females, with onset commonly occurring after the age of 40.^2^ The early stages of EC are often asymptomatic.^3^ However, as the esophageal lumen narrows to less than 14 mm, patients progressively experience dysphagia, initially with solid foods, then semisolids, and eventually liquids or saliva. In China, approximately 70% of patients with EC are diagnosed in the middle or late stages, missing the opportunity for radical surgical intervention.^4^

For inoperable patients, the treatment options include radiotherapy, chemotherapy, synchronous chemoradiotherapy, targeted therapy, and immunotherapy.^5^ Over 95% of ECs in China are squamous cell carcinomas, which are relatively sensitive to radiation^4^, thus underscoring the critical role of radiotherapy in comprehensive EC treatment.^6^ Advancements in technology, such as intensity-modulated radiotherapy (IMRT), image-guided radiation therapy (IGRT), volumetric arc therapy (VMAT), and proton therapy, have significantly improved the management of advanced EC.^7^

The search for molecular biomarkers to predict EC prognosis continues, with researchers facing challenges such as the high cost of testing reagents and the difficulty in obtaining specimens.^8^ Recently, routine blood tests, such as the neutrophil–lymphocyte ratio (NLR), have gained attention for their prognostic value in various solid tumors, including head and neck, nonsmall cell lung, and cervical cancers, and their association with poor EC outcomes.^9,10^ This study specifically examined peripheral blood immune cell subpopulations, T cells, B cells, and natural killer (NK) cells to assess their relationships with EC prognosis. The analysis of these immune cell ratios and activities could provide insights into the body’s immune function.

We retrospectively analyzed data from 72 patients with EC treated at Jiangsu University Hospital to explore the correlation between peripheral blood immune cell subsets and patient outcomes. The results of this study are detailed below.

## Patients and methods

### Patients of the study

This study included 72 patients with esophageal cancer at the Affiliated Hospital of Jiangsu University from December 2021 to December 2023. The cohort comprised 48 males and 24 females, with a median age of 64 years (range: 52–98 years). The majority (68 patients) presented with squamous cell carcinoma of the esophagus, while 3 had small cell carcinoma, and 1 was diagnosed with adenocarcinoma. All individuals underwent a pathological examination at Jiangsu University Hospital, confirming advanced esophageal cancer and missing the optimal window for surgical intervention. Pathological diagnoses were conducted according to the 2019 World Health Organization (WHO) revised classification standards for digestive system tumors.^11^

The inclusion criteria were as follows: (1) pathologically confirmed malignant tumors of the esophagus; (2) assessment of absolute lymphocyte subpopulation counts; (3) computed tomography (CT) scans conducted within one month prior to treatment and three months postradiotherapy; and (4) completion of the planned radiotherapy regimen. The exclusion criteria included the following: (1) patients unsuitable for treatment initiation; (2) patients with nonevaluable lesions; (3) patients with concomitant severe infections or autoimmune diseases; (4) patients with a history of other diagnosed tumors; (5) patients with poorquality CT images with interfering artifacts; (6) patients with concurrent severe medical conditions such as cardiac insufficiency or hepatic and renal dysfunction; (7) patients with cognitive or psychiatric disorders; and (8) patients with a history of drug abuse or alcoholism. This study adhered to the ethical guidelines of the World Medical Association’s Declaration of Helsinki, which was revised in 2013.^12^ The study was approved by the Ethics Committee of the Affiliated Hospital of Jiangsu University. Samples were taken from patients after providing informed consent and with the approval of the Affiliated Hospital of Jiangsu University Ethics Committee (Ethical approval number: KY2021K0902).

### Radiotherapy methods

A thermoplastic body membrane was used to immobilize the patients during treatment. Positioning was further refined via CT-enhanced scans. All the patients underwent IMRT, IGRT, or VMAT. Enhanced CT localization scans with a slice thickness of 5 mm were performed in the supine position, and the resulting imaging data were integrated into the radiation treatment planning system.

At least two clinicians delineated the gross target volume (GTV), nodal GTV (GTVnd), and critical organs at risk, such as the spinal cord and lungs, via upper gastrointestinal imaging and enhanced CT scans. The clinical target volume (CTV) was defined by expanding the GTV laterally by 0.5 cm and vertically by 2.5 cm. Similarly, the GTVnd expanded laterally by 0.5 cm and vertically by 0.5 cm. The planning target volume (PTV) was established by extending the CTV by 0.5 cm in all directions. The prescribed radiation dose for the PTV was set at 60-70 Gy, delivered in 30 fractions over 6–7 weeks, with a frequency of 5 sessions per week, ensuring that 95% of the PTV received the prescribed dose.

### Diagnostic and staging methods

Upon admission, each patient underwent a series of diagnostic procedures to determine the extent and location of disease invasion. These included endoscopy with biopsy, CT, gastrointestinal imaging, or positron emission tomography-computed tomography. The diagnosis of EC was established on the basis of the WHO histological classification, referencing the 2019 edition of the WHO classification of tumors of the gastrointestinal system, and aligned with the Tumor Node Metastasis (TNM) staging system for EC (8^th^ edition, American Joint Committee on Cancer (AJCC); 2017).

The Response Evaluation Criteria in Solid Tumors were applied for staging and ongoing evaluation. These criteria classify the responses of patients with EC into four categories: complete response (CR), partial response (PR), stable disease (SD), and progressive disease (PD).^13^ The key endpoints of the study included overall survival (OS) and progression-free survival (PFS).

### Clinical data collection

This study employed a retrospective design to gather comprehensive data from patients treated for EC. The collected data included general clinical information, laboratory test results, and imaging outcomes. The specific parameters documented were age, sex, TNM stage, complete blood count, absolute lymphocyte subpopulation count, Eastern Cooperative Oncology Group (ECOG) performance status, and other relevant clinical characteristics. The primary endpoint was the OS of patients who underwent radiotherapy for EC. The protection measures of clinical data in this study mainly include data encryption, data anonymisation and access control. These measures ensure the security of data during transmission and storage while protecting individual privacy. Researchers, healthcare providers and data managers can access the corresponding data, but all are subject to strict privilege management and ethical review. With these combined measures, the security and privacy of clinical data can be effectively protected. All procedures conducted in this study adhered to the ethical standards of the 2013 revised World Medical Association (WMA) Declaration of Helsinki.

### Calculation of the neutrophil-to-CD4^+^ T-cell ratio (N/CD4) and the neutrophil-to-CD8^+^ T-cell ratio (N/CD8)

After EDTA-blood samples from patients were collected, 100 μL of each blood sample were used for flow cytometry analysis. In brief, red blood cells were lysed with 2 ml lysing solution for 10 min. The remaining cells were washed twice in wash buffer and then stained with a cell viability dye in PBS for 15 min at room temperature. Cell surface staining was performed with in a cocktail of antibodies, including anti-human CD3, CD4, CD8, CD45RO, and CCR7, in instructed dilutions for 25 min at 4°C. The cells were then fixed and permeabilized with a kit and intracellular staining of FOXP3 was performed. After staining, the whole single-cell suspension of each sample was aspired and analyzed flow cytometry (BD FACS Canto II) till the FACS tubes were empty.^14^ N/CD4 and N/CD8 were calculated on the basis of the absolute neutrophil count (N) and absolute lymphocyte count (L) from the patient’s routine blood test results at the time of initial diagnosis via the following formulas: N/CD4 = absolute neutrophil count (× 10^9^/L)/absolute CD4^+^ absolute T-lymphocyte count (× 10^9^/L); N/CD8 = absolute neutrophil count (× 10^9^/L)/absolute CD8^+^ absolute T-lymphocyte count (× 10^9^/L).

### Follow-up and prognostic analysis

In this study, patient follow-up was conducted primarily through reviews of hospitalization records and outpatient visits. Telephone follow-up was conducted for unresolved or overlooked issues. These follow-ups were scheduled quarterly, with a final cutoff date of December 31, 2023. PFS was measured from the time of disease diagnosis until disease progression or last follow-up contact, whether by phone or outpatient visit. OS was defined as the time from diagnosis to death from any cause or until final follow-up.

### Methods of statistical analysis

Statistical analysis of the data was performed via Statistical Product and Service Solutions (SPSS) (version 26.0) software. Receiver operating characteristic (ROC) curves were generated to evaluate the diagnostic performance of N/CD4 and N/CD8, with OS status serving as the state variable. The area under the curve (AUC) was calculated for each sample, with values greater than 0.5 indicating significant discriminative ability. The optimal cutoff values for N/CD4^+^ T cells and N/CD8^+^ T cells were determined on the basis of the maximal Youden index.

Demographic and clinical characteristics, including sex, age, TNM stage, pathological type, and ECOG score, were categorized and analyzed. Categorical data are expressed as percentages and were analyzed via the χ^2^ test for intergroup comparisons. The Kaplan–Meier method was utilized to construct survival curves for patients categorized into low and high groups on the basis of test variables, and differences in survival were assessed via the log-rank test. Influential factors, such as sex, age, TNM stage, pathological type, ECOG score, N, L, NLR, CD4^+^ T lymphocytes, CD8^+^ T lymphocytes, B cells, and NK cells, were included in the univariate analyses. Factors that reached statistical significance in these analyses were further evaluated via a multivariate Cox proportional hazard regression model to assess their impact on survival outcomes. All the statistical tests were two-sided, with *p* < 0.05 considered statistically significant.

## Results

### General clinical information

Among the 72 patients in this study, 48 were male and 24 were female, with a median age of 64 years (52–98 years). Sixty-one patients (84.7%) were aged > 64 years. Sixteen (22.2%) patients had a history of smoking. Seventeen (23.6%) patients had a history of alcohol consumption. The pathological types were as follows: 68 cases (94.4%) of squamous carcinoma, 1 case (1.4%) of adenocarcinoma, and 3 cases (4.2%) of small-cell carcinoma; 10 cases (13.9%) were poorly differentiated, 51 cases (70.8%) were moderately differentiated, and 11 cases (15.3%) were highly differentiated. The lesions were located in the upper segment in 15 patients (20.8%), the middle segment in 36 patients (50.0%), and the lower segment in 21 patients (29.2%). The T stage was I–II in 30 patients (41.7%). The N stage was 0 for 29 patients (40.3%). An ECOG score of 0 was observed in 44 patients (61.1%), 1 in 22 (30.6%), and 2 in 6 (8.3%). There were 40 patients (55.6%) with malnutrition, 21 patients (29.2%) with anemia, and 9 patients (12.5%) with coagulation abnormalities (Table 1).

**TABLE 1.** Basic physiological and physiological characteristics of 72 patients

Characteristic	No of patents (%)
All patients (%)	72 (100%)
Sex	
Female	24 (33.3%)
Male	48 (66.7%)
Age	
Mean-SD	64 years
Range	52-98
64-year old or older	61 (84.7%)
Under 64-year old	11 (15.3%)
History of smoking	
Yes	16 (22.2%)
No	56 (77.8%)
Drinking history	
Yes	17 (23.6%)
No	55 (76.4%)
Differentiation	
Highly differentiation	11 (15.3%)
Medium differentiation	51 (70.8%)
Low differentiation	10 (13.9%)
Tumor site	
Upper thoracic portion	15 (20.8%)
Middle thoracic portion	36 (50.0%)
Low thoracic portion	21 (29.2%)
Histology	
Squamous	68 (94.4%)
Non-squamous	1 (1.4%)
Small cell	3 (4.2%)
T-staging	
T1 + T2	30 (41.7%)
T3 + T4	42 (58.3%)
N-staging	
N0	29 (40.3%)
N1 + N2	43 (59,7%)
Nutriture	
Benign	32 (44.4%)
Unbenign	40 (55.6%)
Anemic state	
Not anemic	51 (70.8%)
Anemia	21 (29.2%)
Coagulation state	
Normal	63 (87.5%)
Abnormal	9 (12.5%)
ECOG score	
0 point	44 (61.1%)
1 point	22 (30.6%)
2 point	6 (8.3%)

1 ECOG = Eastern Cooperative Oncology Group; SD = standard deviation

### Univariate Cox regression analysis of prognosis in patients with EC

To explore the relationship between patients’ general clinical information and peripheral blood lymphocyte subpopulations and the prognosis of patients with EC undergoing radiotherapy and to remove some irrelevant predictor variables, we performed a one-way analysis of the collated data via SPSS. The results of the prognostic univariate analysis of patients with EC revealed that the ECOG score, CD4^+^ T lymphocytes, N/CD4^+^ T lymphocytes, N/CD8^+^ T lymphocytes, and neutrophil-to-B-cell ratio (N/B) were correlated with the survival of patients who were treated with definitive radiotherapy for EC (*p*< 0.05). The results of the prognostic univariate analysis of patients with EC are as follows (Table 2).

**TABLE 2.** Univariate Cox regression analysis of the relationship between pathophysiological parameters and survival time of patients

Parameter	95% CI	*P*-value
Sex	0.5551–0.7782	0.863
Age	0.7621–0.9324	0.424
Smoking history	0.1238–0.3206	0.150
Drinking history	0.1356–0.3366	0.073
Differentiation	1.8861–2.1416	0.876
Tumor site	1.9172–2.2495	0.977
Histology	0.9993–1.1951	0.150
T-staging	1.4667–1.7000	0.484
N-staging	0.4812–0.7133	0.644
ECOG score	0.3196–0.6248	0.000
Lymphocytes	1.1019–1.3953	0.690
Neutrophils	4.5463–6.0399	0.101
CD4	494.9039–630.8183	0.003
CD8	318.8047–410.5008	0.099
B	127.2621–174.9045	0.441
NK	311.61–429.0567	0.345
CD4/CD8	1.5587–1.9595	0.380
NLR	4.11727–5.9177	0.219
N/CD4	0.00989131–0.01451622	0.000
N/CD8	0.01492113–0.02114241	0.000
N/B	0.04171439–0.07256377	0.020
N/NK	0.01579055–0.02567177	0.141

1 B = B cell; CD4 = CD4^+^ T-cell; CD8 = CD8^+^ T-cell; CI = confidence interval; ECOG = Eastern Cooperative Oncology Group; NK = natural killer cells; N = neutrophil cells; N/CD4 = neutrophil-to-CD4^+^ T-cell ratio; N/NCD8 = neutrophil-to-CD8^+^ T-cell ratio; NLR = neutrophil-lymphocyte ratio

### Multivariate Cox regression analysis of the prognosis of patients with EC

On the basis of the results of univariate analyses of prognosis in patients with EC, we performed multivariate analyses to disentangle the effects of other confounders further to determine the correlation between predictor variables and the prognosis of patients with EC. The results of the multifactorial analysis of the prognosis of patients with EC revealed that N/CD4 (hazard ratio [HR] = 4.14474183122331E^+73^, *p* = 0.009) and N/CD8 (HR = 4.26629029136702E^-41^, *p* = 0.021) were independent risk factors affecting the prognosis of patients with EC (Table 3).

**TABLE 3.** Multivariate Cox regression analysis of the relationship between clinical variables and patient survival

Parameter	HR	95% CI Lower	95% CI Upper	*P*-value
N/CD4	4.14E^+73^	3.39128E^+18^	5.07E^+128^	0.009
ECOG	2.151826047	1.202835884	3.849532092	0.010
N/CD8	4.27E^-41^	2.01E^-75^	9.04E^-07^	0.021
N/B	0.00138624	6.32E^-12^	303904.1296	0.502
CD4	0.999658052	0.997587163	1.00173324	0.747

1 B = B cells; CD4 = CD4^+^ T-cell; CI = confidence interval; HR = hazard ratio; N = neutrophil cells; N/CD4 = neutrophil-to-CD4^+^ T-cell ratio; N/NCD8 = neutrophil-to-CD8^+^ T-cell ratio

### ROC curves

**Figure 1.**
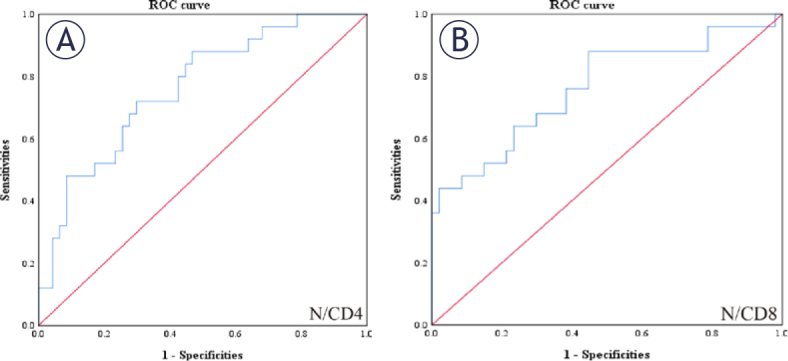
Receiver operating characteristics (ROC) curve plotted to determine the value of a statistically significant variable in the Cox regression model for neutrophil-to-CD4^+^ T-cell ratio (N/CD4) **(A)** and neutrophil-to-CD8^+^ T-cell ratio (N/CD8) **(B)** according to ROC analysis, the area under the curve of N/CD4 and N/CD8 was 0.763 and 0.763, respectively, and the optimal cutoff point was 0.01053329 and 0.01184294, respectively.

In the dataset, we find class imbalances when there are many more negative samples than positive samples (or vice versa), and the distribution of positive and negative samples in the data may also change over time. When the distribution of positive and negative samples in the data changed, the ROC curve remained unchanged. However, the ROC curve does not clearly indicate which variable is more effective. The AUC is defined as the area under the ROC curve as a numerical value; its value range is generally between 0.5 and 1, which clearly and intuitively indicates that the indicator is better. Therefore, we used the AUC as an evaluation criterion. The best cutoff value for N/CD4 was obtained by the ROC curve for N/CD4^+^ T cells and was 0.01053329, with a sensitivity of 0.72, specificity of 0.702, and AUC of 0.763 (95% confidence interval [CI]: 0.649–0.876) (Figure 1A). The best cutoff value of N/CD8 was obtained by the ROC curve of N/CD8 as 0.01184294, with a sensitivity of 0.88, specificity of 0.553, and AUC of 0.763 (95% CI: 0.640–0.886) (Figure 1B).

### Survival analysis of patients with EC stratified by N/CD4^+^ T lymphocytes and N/CD8^+^ T lymphocytes

The median follow-up time of the 72 patients with EC in this study was 12 months (0–25 months). The median OS time for patients in the low- and high-N/CD4 group were 372 and 750 days, respectively. The 1-year OS rate of patients in the low N/CD4 group was also significantly greater than that of patients in the high N/CD4 group (HR = 18.15, 95% CI: 2.44–134.90, *p*< 0.05) (Figure 2A). The median OS times of patients in the low- and high-N/CD8^+^ T-cell groups were 751 and 310 days, respectively. Moreover, the 1-year OS rate of patients in the low-N/CD8 group was significantly lower than that of patients in the high-N/CD8 group (HR = 3.39, 95% CI: 1.43–8.00, *p*< 0.05) (Figure 2B).

**Figure 2A:**
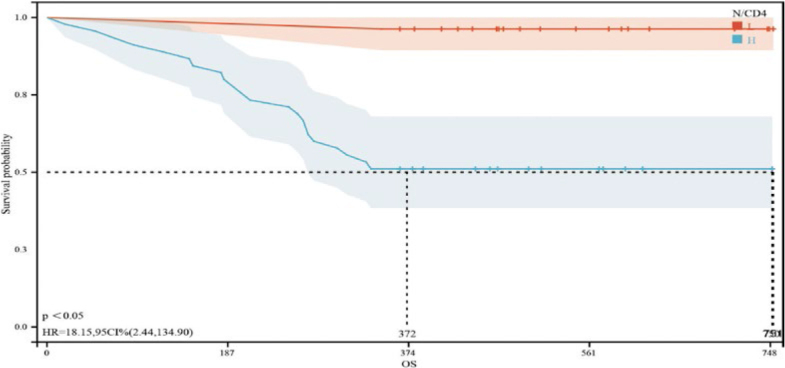
Kaplan-Meier survival curves for patients with advanced oesophageal cancer in different neutrophil-to-CD4^+^ T-cell ratio (N/CD4) groups. The red curve represents the overall survival of patients with an N/CD4 less than 0.01053329, while the blue curve represents the overall survival of patients with an N/CD4 greater than or equal to 0.01053329. The mean survival time of patients in the low- and hight-N/CD4 group were 372 and 750 days, respectively, with a p < 0.05, indicating a significant difference between the two groups.

**Figure 2B:**
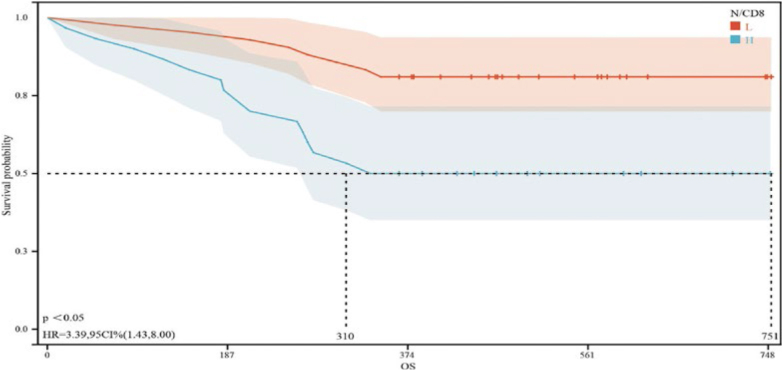
Kaplan-Meier survival curves for patients with advanced cancer in different neutrophil-to-CD4^+^ T-cell ratio (N/CD4) groups. The red curve represents the overall survival of patients with an N/CD4 less than 0.01184294, while the blue curve represents the overall survival of patients with an N/CD4 greater than or equal to 0.01184294. The mean survival time of patients in the low- and high-N/CD4 group were 751 and 310 days, respectively, with a p < 0.05, indicating a significant difference between the two groups.

### Relationships between patients’ clinical characteristics and N/CD4^+^ T cells or N/CD8^+^ T cells

According to the optimal cutoff value of N/CD4, 40 patients were included in the low N/CD4 group (N/CD4 < 0.01053329), and 32 patients were included in the high N/CD4 group (N/CD4 ≥ 0.01053329). The differences in tumor location and anemia status between the two groups were statistically significant (*p*< 0.05). However, the differences in the other clinical characteristics were not statistically significant (*p*> 0.05) (Table 4). According to the optimal cutoff value of N/CD8, 29 patients were included in the low N/CD8 group (N/CD4 < 0.01184294), and 43 patients were included in the high N/CD8 group (N/CD8 ≥ 0.01184294). The tumor location, tumor differentiation, ECOG score, and RT efficacy of the patients in the two groups were compared. Furthermore, the differences were statistically significant (*p*< 0.05), and no statistically significant differences were found when other clinical characteristics were compared (*p*> 0.05) (Table 5).

**TABLE 4.** Association of pathological features and neutrophil-to-CD4^+^ T-cell ratio (N/CD4) in patients

Characteristic, n = 72	N/CD4 ≤ 0.01053329	N/CD4 > 0.01053329	*P-*value
Sex			
Female	12	12	
Male	28	20	0.502
Age			
≥ 64 years old	33	28	
< 64 years old	7	4	0.558
History of smoking			
Yes	8	8	
No	24	32	0.612
Drinking history			
Yes	10	7	
No	30	25	0.756
Differentiation			
Highly differentiation	6	5	
Medium differentiation	29	22	
Low differentiation	5	5	0.228
Tumor site			
Upper thoracic portion	9	6	
Middle thoracic portion	23	13	
Low thoracic portion	8	13	0.032
Histology			
Squamous	36	32	
Nonsquamous	1	0	
Small cell	3	0	0.368
T-staging			
T1 + T2	15	15	
T3 + T4	25	17	0.423
N-staging			
N0	19	10	
N1 + N2	21	22	0.162
Nutriture			
Benign	19	13	
Unbenign	21	19	0.56
Anemic state			
Not anemic	7	15	
Anemia	33	17	0.007
Coagulation state			
Normal	30	23	
Abnormal	10	9	0.765
ECOG score			
0 point	24	20	
1 point	11	11	
2 point	5	1	0.366
Treatment efficacy			
CR + PR	26	21	
SD + PD	14	11	0.956

1 CR = complete response; ECOG = Eastern Cooperative Oncology Group; PR = partial response; SD = stable disease; PD = progressive disease

**TABLE 5.** Association of pathological features and neutrophil-to-CD8^+^ T-cell ratio (N/CD8) in patients

Characteristic, n = 72	N/CD8 ≤ 0.01184294	N/CD8 > 0.01184294	*P*-value
Sex			
Female	9	15	
Male	20	28	0.734
Age			
≥ 64 years old	26	35	
< 64 years old	3	8	0.339
History of smoking			
Yes	6	10	
No	23	33	0.797
Drinking history			
Yes	6	11	
No	23	32	0.632
Differentiation			
Highly differentiation	5	6	
Medium differentiation	19	32	
Low differentiation	5	5	0.040
Tumor site			
Upper thoracic portion	4	11	
Middle thoracic portion	15	21	
Low thoracic portion	10	11	0.047
Histology			
Squamous	27	41	
Nonsquamous	1	0	
Small cell	1	2	0.802
T-staging			
T1 + T2	12	18	
T3 + T4	17	25	0.968
N-staging			
N0	13	16	
N1 + N2	16	27	0.518
Nutriture			
Benign	15	17	
Unbenign	14	26	0.307
Anemic state			
Not anemic	7	15	
Anemia	22	28	0.332
Coagulation state			
Normal	20	33	
Abnormal	9	10	0.463
ECOG score			
0 point	22	22	
1 point	7	15	
2 point	0	6	0.035
Treatment efficacy			
CR + PR	26	21	
SD + PD	3	22	0.000

1 CR = complete response; ECOG = Eastern Cooperative Oncology Group; PR = partial response; SD = stable disease; PD = progressive disease

## Discussion

EC is a malignant tumor originating from the epithelial cells of the esophagus. The primary clinical manifestations of EC include choking while swallowing food, the sensation of a foreign body in the throat, retrosternal pain, and significant dysphagia. Complications can escalate when the tumor metastasizes to or invades nearby organs, leading to pain and discomfort in the affected areas. Statistically, 70% of EC cases in China are diagnosed at a middle or late stage, which often precludes curative surgical resection.^4^ Predominantly, these cancers are diagnosed as squamous cell carcinomas, accounting for more than 95% of EC cases in the region.^4^ This subtype is notably sensitive to radiation, underscoring the need for a comprehensive treatment strategy that typically combines preoperative radiotherapy with surgical intervention or intensive radiochemotherapy to increase patient survival.^5^

The ongoing research and development of molecular biomarkers have become crucial in predicting the prognosis of patients with EC.^8^ However, the use of these biomarkers in clinical practice is challenging because of the high costs and logistical difficulties associated with obtaining and processing the necessary test samples. Despite these hurdles, the identification of more effective and accessible biomarkers is critical. These biomarkers could significantly improve prognostic accuracy and help tailor individualized treatment plans for patients, thereby potentially improving the overall outcomes of EC management. As challenges in the discovery of reliable and accurate biomarkers, we consider the following points in our future research:

1. Standardised methods: inter- and intra-variability can be significantly reduced by standardised experimental and analytical methods, improving the reliability and reproducibility of biomarkers.^15^
2. Multi-centre studies: multi-centre studies can better assess the performance of biomarkers in different populations and reduce the variability caused by individual differences, thus increasing the value of their clinical application.^16^
3. Repeated measurements: repeating measurements several times and using statistical methods to assess consistency can more accurately assess the performance of biomarkers.^17^
4. Quality control: strict quality control measures can reduce technical variability, including the use of internal standards and regular calibration of instruments, thus improving the reliability of results.^18^

In recent years, routine blood tests have become the cornerstone of clinical protocols for diagnosing and managing malignant tumors, playing a crucial role in treatment guidance and patient outcome assessment. Among the metrics derived from these tests, the NLR has been identified as a significant predictor of prognosis across various solid tumors, including head and neck cancer, non-small cell lung cancer, and breast cancer.^9,10^ Studies such as those conducted by Shao *et al*.^19^ and Shao *et al*.^20^ A high NLR often correlates with a poor prognosis, establishing an elevated NLR as an independent risk factor in patients with lung cancer (OS: 22.0 months *vs*. 11.7 months, HR = 1.6, *p* = 0.001; PFS: 11.1 *vs*. 6.0 months, *p*< 0.001)^19^ and a lower NLR as an independent protective factor in patients with breast cancer (PFS: 14.8 *vs*. 7.2 months, HR =1.791, *p* = 0.003; OS: 64.1 *vs*. 56.0 months, *p* = 0.980).^20^

The utility of the NLR extends to EC, which is strongly associated with adverse outcomes. However, the specific immune cells contributing to these effects remain underexplored, partly because lymphocytes, the “L” in the NLR, constitute numerous subgroups, and few studies have applied these subgroups to the prognosis of patients with EC. Typically, the balance between various lymphocyte subpopulations, including T cells, B cells, and NK cells, is critical for maintaining and regulating the immune functions of the body. Accordingly, analyzing the ratios and activities of these cells is essential for evaluating their immune competence.

This study retrospectively analyzed the data of 72 patients with EC from Jiangsu University Hospital to explore the relationships between peripheral blood immune cell subsets and EC prognosis. Our findings suggest that the relationship between the NLR and RT sensitivity in patients with EC might vary, possibly because different cutoff values are applied across diverse patient groups and geographical regions. Univariate analysis revealed that factors, including the ECOG performance score and the ratios of CD4^+^ T lymphocytes, N/CD4^+^ T lymphocytes, N/CD8^+^ T lymphocytes, and N/B cells, were significantly correlated with the survival outcomes of patients who underwent definitive radiotherapy for EC (*p*< 0.05).

Furthermore, the multifactorial analysis identified elevated ratios of N/CD4 (HR = 4.14474183122331E^+73^, *p* = 0.009) and N/CD8 (HR = 4.26629029136702E^-41^, *p* = 0.021) as independent risk factors that adversely affect EC prognosis. This insight allows clinicians to use the N/CD4 and N/CD8 ratios to refine prognostic evaluations and optimize treatment plans for patients with EC undergoing radiation therapy. ROC curve analysis revealed that the optimal cutoff value for N/CD4 was 0.01053329 (with a sensitivity of 0.72, specificity of 0.702, and AUC of 0.763, 95% CI: 0.649–0.876) and that for N/CD8 was 0.01184294 (with a sensitivity of 0.88, specificity of 0.553, and AUC of 0.763, 95% CI: 0.640–0.886). Subgroup analysis on the basis of these cutoff values revealed statistically significant differences in tumor location, anemia status, degree of tumor differentiation, ECOG score, and efficacy of radiation therapy between the groups with low and high N/CD4 and N/CD8 ratios, confirming the practical value of these biomarkers in clinical settings.

In conclusion, the results of this study suggest that low N/CD4 and N/CD8 ratios are independent risk factors affecting the prognosis of patients with EC and that N/CD4 and N/CD8 ratios, which are inexpensive and easily accessible clinical indicators, may have predictive value for the prognosis of patients with EC. However, this study is a smallsample, single-center, retrospective observational study, which still needs to be validated by further large-sample prospective studies.
